# DNA methylation and hydroxymethylation analyses of the active LINE-1 subfamilies in mice

**DOI:** 10.1038/s41598-017-14165-7

**Published:** 2017-10-19

**Authors:** Yui Murata, Miki Bundo, Junko Ueda, Mie Kubota-Sakashita, Kiyoto Kasai, Tadafumi Kato, Kazuya Iwamoto

**Affiliations:** 1 Department of Molecular Brain Science, Graduate School of Medical Sciences, Kumamoto University, 1-1-1 Honjo, Chuo-ku, Kumamoto City, Kumamoto, 860-8556 Japan; 2 PRESTO, Japan Science and Technology Agency, 4-1-8 Honcho, Kawaguchi City, Saitama, 332-0012 Japan; 3 Laboratory for Molecular Dynamics of Mental Disorders, RIKEN Brain Science Institute, 2-1 Hirosawa, Wako-city, Saitama, 351-0198 Japan; 4 Department of Neuropsychiatry, Graduate School of Medicine, The University of Tokyo, 7-3-1 Hongo, Bunkyo-ku, Tokyo, 113-8655 Japan

## Abstract

Retrotransposon long interspersed nuclear element-1 (LINE-1) occupies a large proportion of the mammalian genome, comprising approximately 100,000 genomic copies in mice. Epigenetic status of the 5′ untranslated region (5′-UTR) of LINE-1 is critical for its promoter activity. DNA methylation levels in the 5′-UTR of human active LINE-1 subfamily can be measured by well-established methods, such as a pyrosequencing-based assay. However, because of the considerable sequence and structural diversity in LINE-1 among species, methods for such assays should be adapted for the species of interest. Here we developed pyrosequencing-based assays to examine methylcytosine (mC) and hydroxymethylcytosine (hmC) levels of the three active LINE-1 subfamilies in mice (TfI, A, and GfII). Using these assays, we quantified mC and hmC levels in four brain regions and four nonbrain tissues including tail, heart, testis, and ovary. We observed tissue- and subfamily-specific mC and hmC differences. We also found that mC levels were strongly correlated among different brain regions, but mC levels of the testis showed a poor correlation with those of other tissues. Interestingly, mC levels in the A and GfII subfamilies were highly correlated, possibly reflecting their close evolutionary relationship. Our assays will be useful for exploring the epigenetic regulation of the active LINE-1 subfamilies in mice.

## Introduction

A large proportion of the mammalian genome is occupied by transposons and their related sequences, including a retrotransposon called long interspersed nuclear element-1 (LINE-1). LINE-1 is an approximately 6-kb genomic element, composing approximately 20% of the mammalian genome^1–3^. The total amount of LINE-1 in mouse is estimated to approximately 100,000 copies in mice^4^. A full-length LINE-1 contains 5′ untranslated region (UTR), open reading frame (ORF) 1, ORF2, and 3′-UTR, and it can amplify its copy number in the genome via transcription and reverse transcription through a process called retrotransposition^5,6^.

In humans, only the youngest LINE-1 subfamily, Hs, retains retrotransposition activity^7^. The DNA methylation level of the 5′-UTR of LINE-1 Hs is widely used to estimate the global DNA methylation level in the human genome^8^, and it can be measured in cells or tissues affected by various disorders, such as cancer^9–14^, tumors^15^, heart disease^16^, and neuropsychiatric disorders^17–20^.

The structure of LINE-1 varies considerably across species. In mice, at least three LINE-1 subfamilies (Tf, A, and Gf) retain retrotransposition activities, representing more than 9,000 full-length copies in the mouse genome^4,21–23^. The total number of active LINE-1 elements in mice is more than 10-fold higher compared to humans^24^. In addition, repeat tandems, called monomers, occur within the 5′-UTR, and are not found in the human LINE-1^25^. Therefore, DNA methylation assays developed for the 5′-UTR of human LINE-1 cannot be applied to other species.

We developed pyrosequencing-based assays to examine the levels of DNA methylation and hydroxymethylation in the 5′-UTR of the active LINE-1 subfamilies in mice. Hydroxymethylcytosine (hmC), which is widely distributed across tissues and especially enriched in the brain, is modified from methylcytosine (mC) by the ten-eleven translocation (TET) enzymes^26–28^. Because the widely used sodium bisulfite treatment cannot distinguish between mC and hmC, we used oxidation treatment prior to bisulfite modification^29^. Using the developed assay, we examined cytosine modification status in several adult brain regions (frontal cortex, hippocampus, cerebellum, and basal ganglia) and in nonbrain tissues including tail, heart, testis, and ovary. We observed tissue- and subfamily-specific mC and hmC differences, which suggest complex epigenetic regulation of the active LINE-1 subfamilies in adult tissues in mice.

## Results

### Design of pyrosequencing-based assay

**Figure 1:**
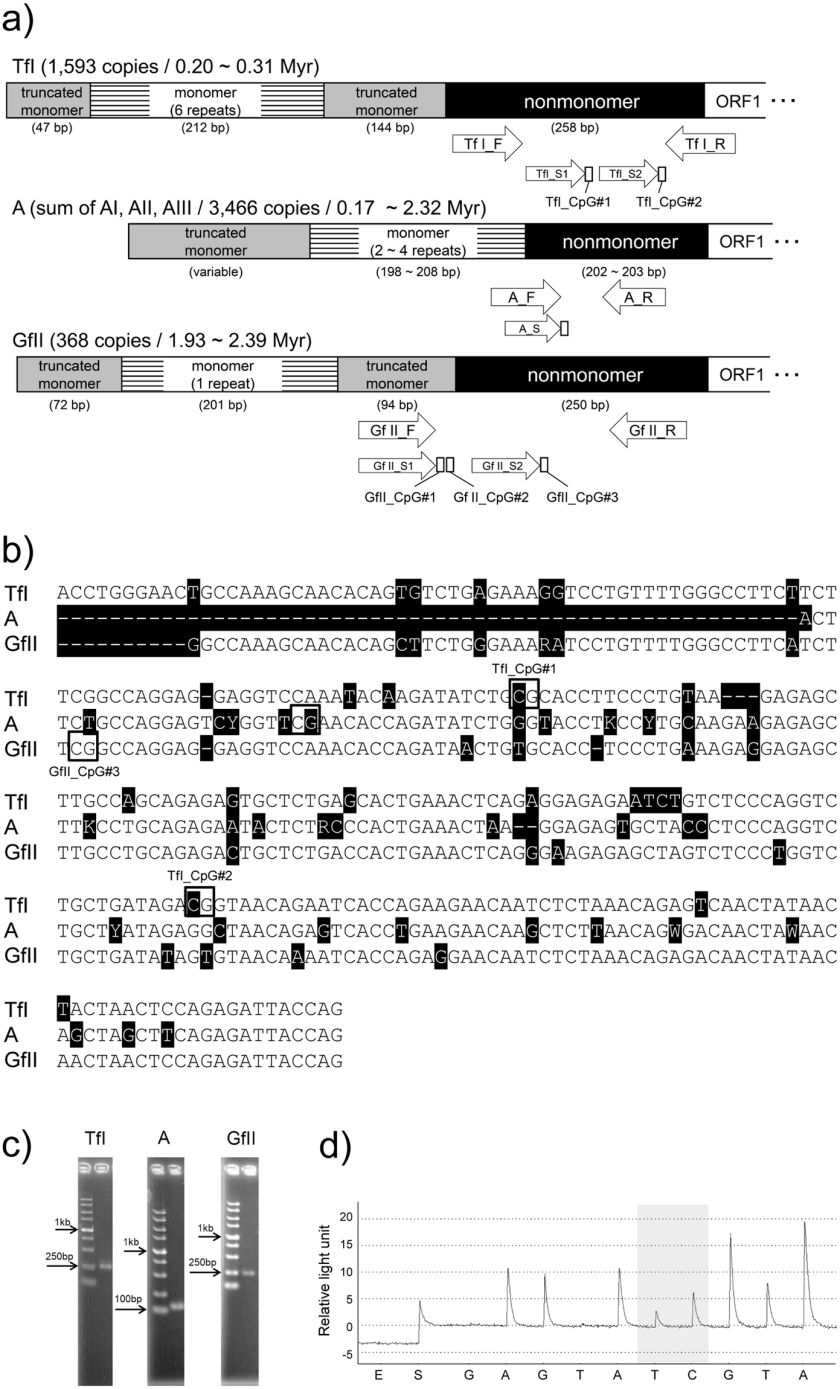
Pyrosequencing-based DNA methylation assay of the active LINE-1 subfamilies in mice. (**a**) Schematic representation of 5′-UTR of the active LINE-1 (TfI, A, and GfII) in mice, drawn from previous studies^23,35^. Designed primers and examined CpG sites are indicated. 5′-UTR of the active LINE-1 subfamilies generally consists of a variable number of monomers and truncated monomers as well as a nonmonomer region upstream of ORF1. Definitions of monomers and nonmonomers were determined previously^23,35^. Numbers in brackets indicate the total copy number of full length elements, and rough age of the subfamily^4^. Myr, million years; ORF, open reading frame; F, forward primer; R, reverse primer; S, sequence primer. (**b**) Alignment of the nonmonomer sequences of TfI, A, and GfII. Nonconserved sequences among these subfamilies are highlighted in black. The analyzed CpGs within the nonmonomer sequences (TfI_CpG#1, TfI_CpG#2, A, and GfII_CpG#3) are boxed. Note that GfII_CpG#1 and GfII_CpG#2 are located in the truncated monomer sequence. (**c**) Agarose gel electrophoresis analysis of the PCR amplicons. The expected amplicon sizes of TfI, A, and GfII are 261 bp, 129 bp, and 244 bp, respectively. A 100-bp DNA ladder (TakaraBio) was used. Arrows indicate respective sizes of bands. (**d**) A representative pyrogram in pyrosequencing analysis (TfI_CpG#2). The pyrosequencing reaction starts with an input of enzyme (E) followed by substrates (S). The sequence after S is as listed as dispensation order in Table 1. The shaded site is the cytosine analyzed in this study.

Consensus sequences of active LINE-1 subfamilies in mice (Tf, A, and Gf), which were characterized in a previous study^4^, were retrieved from Repbase^30,31^. Based on these sequences, we designed PCR primers to the truncated monomer or nonmonomer region of each subfamily for a pyrosequencing assay. We successfully developed the pyrosequencing assay for TfI, A (including AI, AII, and AIII), and GfII (Fig. 1a,b and Table 1). In these assays, we obtained single-banded expected size of PCR amplicons (Fig. 1c), and expected peaks were deduced from the consensus sequence in the pyrogram (Fig. 1d). We confirmed the specificity of each assay by TA-cloning the bisulfite PCR products followed by Sanger sequencing. In the amplification step, these assays showed 95%, 97% and 100% specificity for types TfI, A and GfII, respectively (see Supplementary Figs S1 to S3). Linearity and sensitivity of the assay were assessed using the synthetic DNA samples with different methylation levels. We obtained high correlations (R > 0.998, see Supplementary Fig. S4).

**Table 1:** List of the primers designed, the analyzed sequences, and the dispensation orders.

Subfamily	Primer type	Primer name	Primer sequence	Sequence to analyze	Dispensation order	CpG#
TfI	Forward	Tf I_F	TTTGGGAATTGTTAAAGTAATATAG			
	Reverse	Tf I_R	bio-CCATCTAATAATCTCTAAAATTAATA			
	Sequence 1	Tf I_S1	GGAGGAGGTTTAAAT	ATAAGATATTTG**Y**GTA	TATAGATATAGTCGT	TfI_CpG#1
	Sequence 2	Tf I_S2	TGTTTTTTAGGTTTGTTGAT	AGA**Y**GGTAATAGAATTAT	GAGTATCGTA	TfI_CpG#2
A	Forward	A_F	GTGAGTGGAATATAATTTTTGTTAGGA			
	Reverse	A_R	bio-AAAAAATAACACTCTCCTTAATTTCAATAA			
	Sequence	A_S	GGAATATAATTTTTGTTAGG	AGTTYGGTT**Y**GAATATTAGATATTTGGGTATT	GATGTCAGTCGAT	
GfII	Forward, Sequence 1	Gf II_F, Gf II_S1	GGGGGTTATTTTGATTTTG	GGATTT**Y**GTAG**Y**GGGTA	AGTATTCGTATGTCGT	GfII_CpG#1, CpG#2
	Reverse	Gf II_R	bio-TTCCTCTAATAATTTTATTACACTA			
	Sequence 2	Gf II_S2	TTGTTTTGGGTTTTTATTTT	**Y**GGTTAGGAGG	ATCGTAGA	GfII_CpG#3

The 5′-biotinylated primers are indicated by the “bio-”. Note that one amplicon is analyzed by two different sequencing primers in TfI and GfII. In GfII, the forward primer is also used as a sequencing primer (GfII_S1).

### mC and hmC levels of active LINE-1 subfamilies in mouse tissues

**Figure 2:**
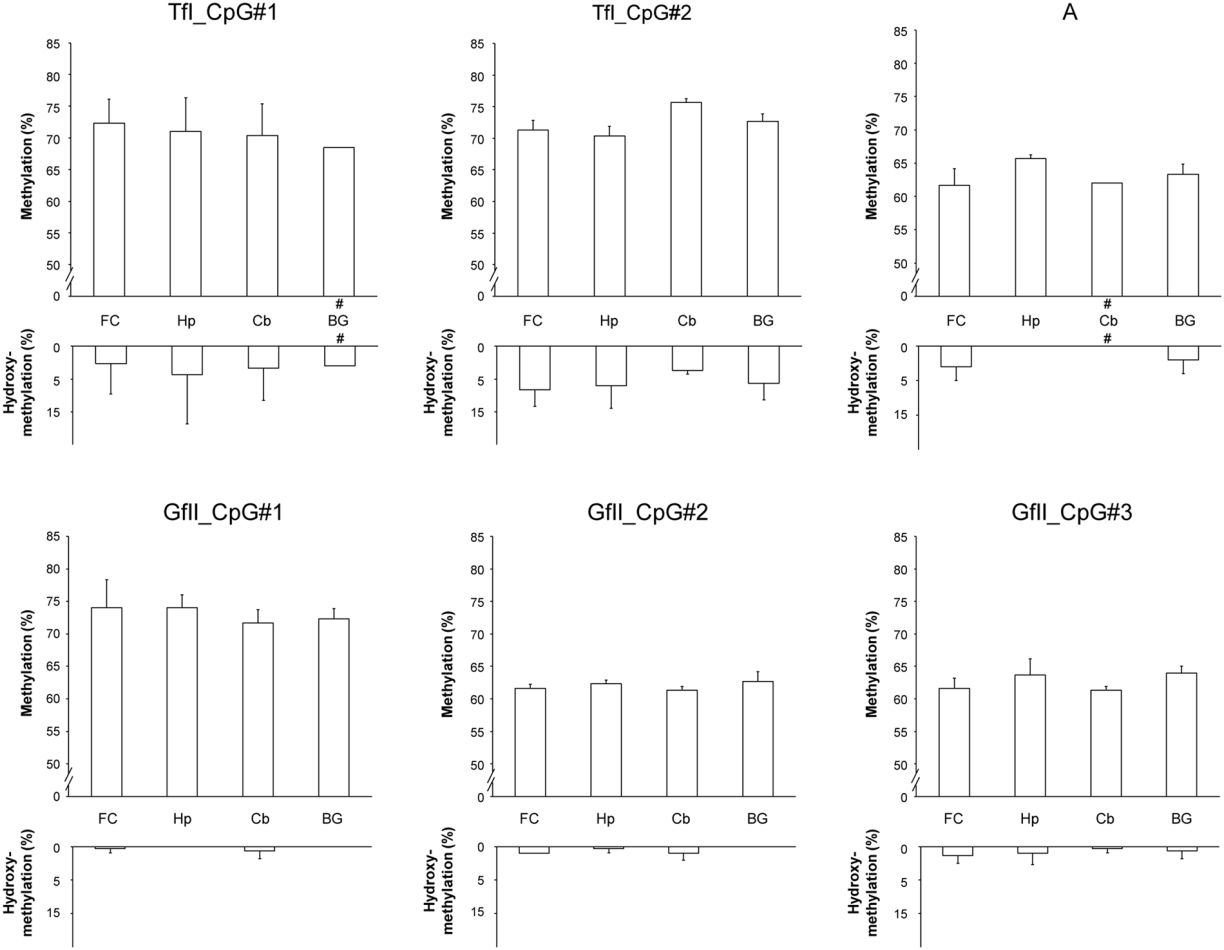
mC and hmC levels of the active LINE-1 subfamilies in the adult mouse brain regions. For TfI, two CpG sites located within the nonmonomer region; for A, one CpG site located within the nonmonomer region; and for GfII, three CpG sites (two located within the truncated monomer, and one within the nonmonomer region) were analyzed. Three samples were available for analysis for each CpG site, except for the samples indicated by # symbol, for which only two were available. Values are given as mean ± standard deviation. mC, DNA methylation; hmC, hydroxymethylation; FC, frontal cortex; Hp, hippocampus; Cb, cerebellum; BG, basal ganglia.

Using the developed assay, we measured mC and hmC levels in various brain and nonbrain tissues of adult mice. By using oxidation reaction before bisulfite modification^29^, the actual mC level at each CpG site was determined. The hmC level could be estimated by subtracting the actual mC level from the bisulfite modification data. We did not find significant differences in mC or hmC levels among the four brain regions, frontal cortex (FC), hippocampus (Hp), cerebellum (Cb), and basal ganglia (BG), using analysis of variance (ANOVA) (Fig. 2). At the subfamily level, we found a relatively high hmC level in the TfI subfamily across all brain regions, while others showed lower or negligible levels, except in the FC and BG in A subfamily.

**Figure 3:**
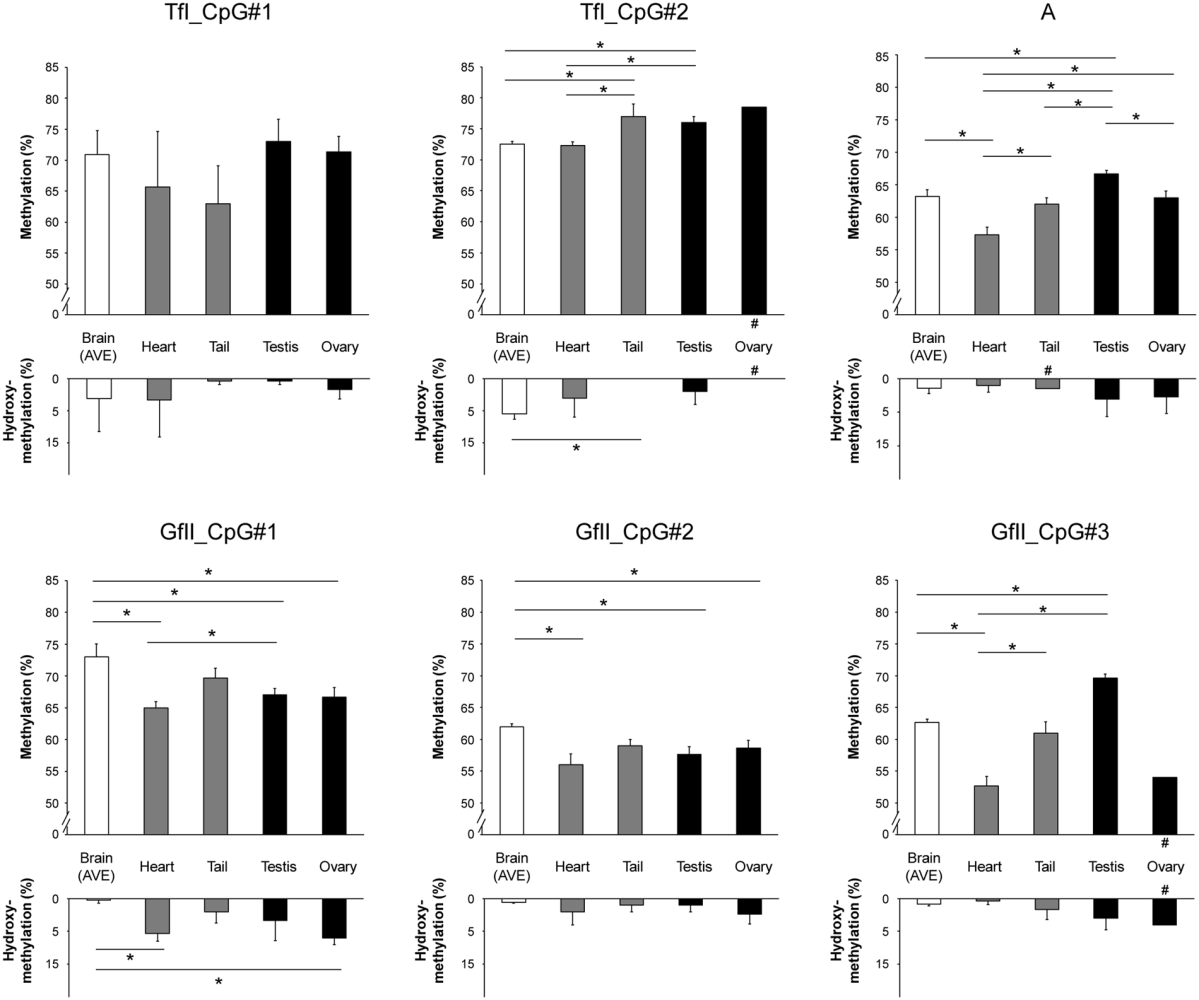
mC and hmC levels of the active LINE-1 subfamilies in the brain and nonbrain tissues. The data from four brain regions were averaged and treated as a single tissue, Brain (AVE). Three samples were available for analysis for each CpG site, except for the samples indicated by # symbol, for which only two were available. Values are given as mean ± standard deviation. *Statistically significant (ANOVA followed by Tukey’s test, *P* < 0.05). mC, DNA methylation; hmC, hydroxymethylation.

In contrast, we observed significant tissue- and subfamily-specific differences in mC and hmC levels in nonbrain tissues (Fig. 3). For TfI (TfI_CpG#2), mC levels of the tail and testis were higher, while hmC levels were lower, compared with the brain and heart. In the nonmonomer regions of A and GfII (GfII_CpG#3), the mC level of the heart was lower and that of the testis was higher compared with the other tissues. In the truncated monomer region of GfII (GfII_CpG#1 and #2), nonbrain tissues showed lower mC and higher hmC levels to a similar extent compared with the brain.

### Correlation of mC levels between tissues or subfamilies

**Figure 4:**
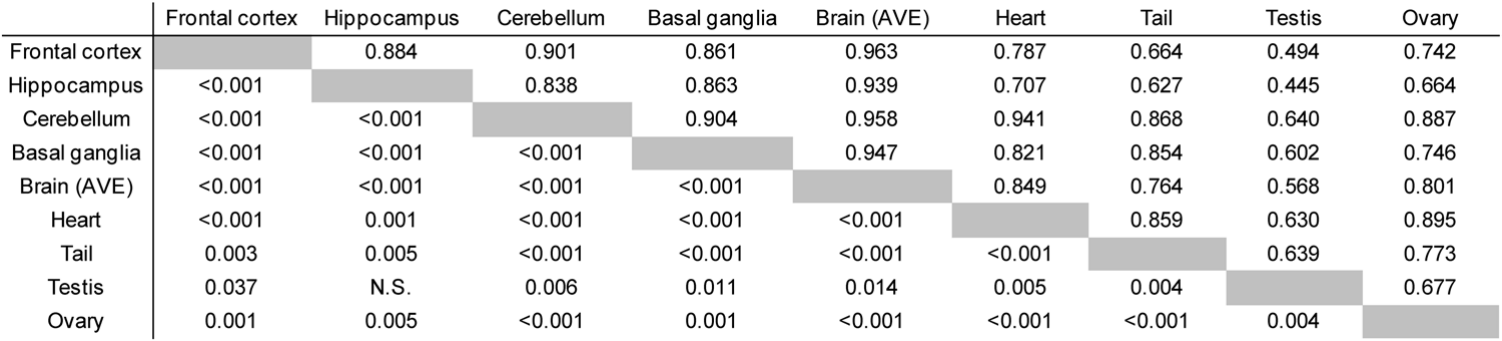
Correlations of actual mC levels of the active LINE-1 subfamilies between two tissues. Pair-wise Pearson’s correlation between two tissues was calculated using actual mC levels of two CpG sites from TfI, one CpG site from A, and three CpG sites from GfII. *R* and *P* values are indicated in upper right and lower left, respectively. Data from four brain regions were averaged and treated as a single brain tissue, Brain (AVE).

**Figure 5:**
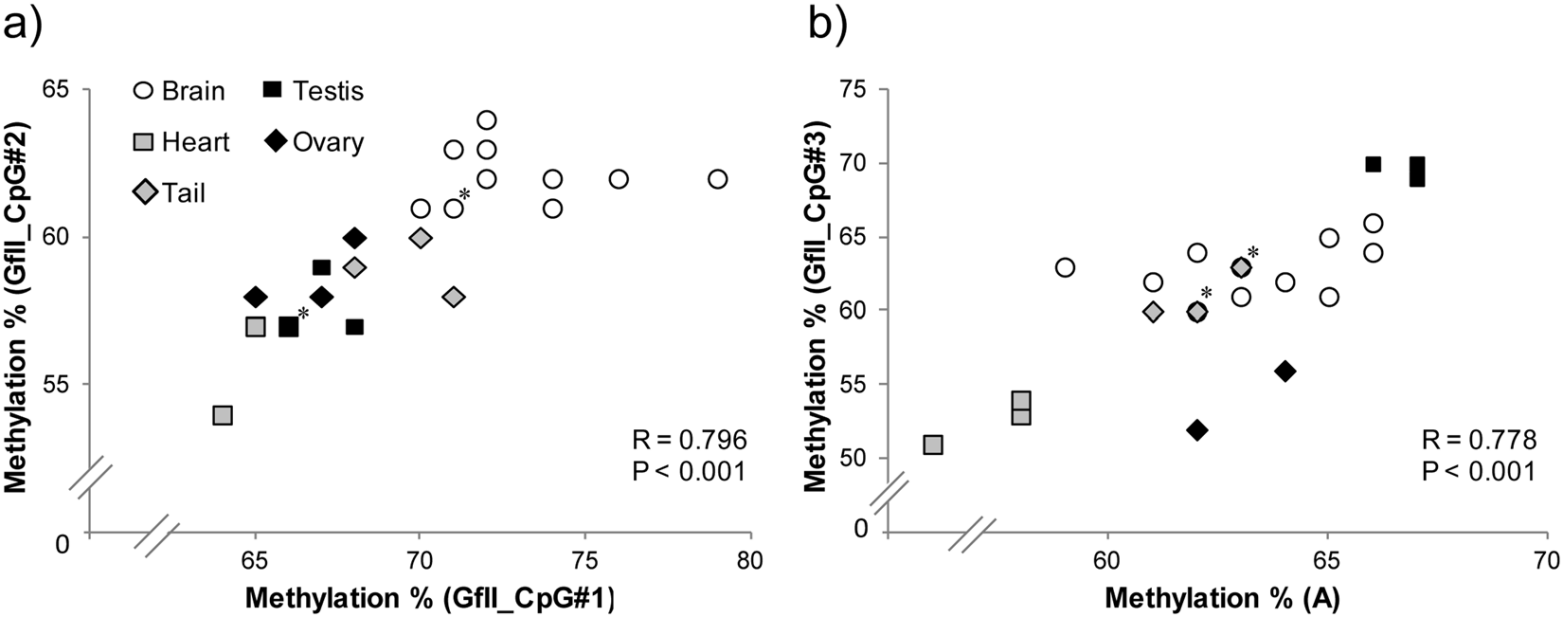
Correlation of actual mC levels between different two CpG sites. Pairs of (**a**) GfII_CpG#1 and GfII_CpG#2, and (**b**) A and GfII_CpG#3 were the only ones that showed significant correlations (*P* < 0.05 after Bonferroni correction). Each symbol represents data from one individual tissue (*N* = 12 for brain regions and *N* = 3 each for other tissues, except for the ovary tissue in GfII_CpG#3, for which only two were available). Symbols with * on the right corner indicate that two samples are overlapped on each other in the graph, because they showed the same DNA methylation levels.

We calculated pair-wise correlations of mC levels across all tissues (Fig. 4). As expected, each brain region showed strong correlations with the other three brain regions (R > 0.8). Interestingly, among the tissues tested, levels in the testis were poorly correlated with those in other tissues. Similarly, we calculated pair-wise correlations of mC levels across all CpG sites (Fig. 5). We found significant strong correlations between CpG sites for GfII_CpG#1 and GfII_CpG#2, as well as between GfII_CpG#3 and A (Fig. 5).

## Discussion

We developed pyrosequencing-based DNA methylation assays of the 5′-UTR in the active LINE-1 subfamilies TfI, A, and GfII in mice, and we evaluated both mC and hmC levels of the brain regions and several other tissues. Given that there are about 1,600, 3,500, and 370 full-length copies in TfI, A, and GfII subfamilies, respectively^4^, the mC and hmC levels obtained with these assays indicate the average values for each subfamily.

Measuring the mC level at the monomer region is difficult, due to the variable number of monomer unit repeats. Therefore, we focused on the CpGs in the nonmonomer or truncated monomer regions. Compared to the monomer regions, the number of CpGs in nonmomer regions seemed to be depleted. Consequently, in TfI, two out of three CpGs in the nonmonomer region were covered by our assay. Although there was only one conserved CpG in the nonmonomer region in both A and GfII, we successfully included these CpGs in the pyrosequencing assays (Fig. 1a,b).

Subfamily- and tissue-dependent epigenetic differences detected in this study suggest that each of the active LINE-1 subfamilies may have distinct roles in the adult brain and other tissues, which have not been well addressed to date. The four brain regions examined showed less variation in mC levels, although subfamily-dependent variations in hmC levels were observed. Among the nonbrain tissues examined, the testis had distinctive epigenetic profiles as exemplified by the low correlation coefficients in comparisons with the other tissues (Fig. 4). This finding might be related to the unique epigenetic regulation of transposons in spermatogenesis^32^. However, the tissues used in this analysis, except for the testis, were derived from female mice. Therefore, our correlation analysis may be affected by the sex difference. Overall, the hmC levels were relatively low (Figs 2 and 3), with individual levels ranging from 0% up to 13%. In general, the brain tissues showed higher hmC levels than the nonbrain tissues for TfI (Fig. 3). In contrast, for GfII, the brain tissues were poorly hydroxymethylated, compared with the other nonbrain tissues.

A strong positive correlation in mC levels was found between GfII_CpG#1 and GfII_CpG#2 (Fig. 5). Since these two CpGs were located within the truncated monomer region (Fig. 1), they are likely to be under the same epigenetic regulation. Unexpectedly, the mC level of A showed a strong correlation with GfII_CpG#3, but not with other CpG sites in GfII (GfII_CpG#1 or GfII_CpG#2) or TfI (TfI_CpG#1 or TfI_CpG#2). This observation may be attributable to their evolutionary relationship. Phylogenetic analysis based on the conserved ORF2 sequences previously revealed that A (AI, AII, and AIII) and GfII are clustered in the same phylogenetic branch^4^. Therefore, epigenetic status of each subfamily may reflect evolutionary aspect, despite that A family consists of A-type monomer and Gf family has F-type monomer, which is distinct from A-type in terms of sequence and structure of monomer region^33^.

The pyrosequencing-based assays we developed would be useful for rapid and accurate high-throughput assessment of epigenetic regulation of the active LINE-1 subfamilies in mice.

## Materials and Methods

### Consensus sequences of mouse LINE-1 elements

We retrieved consensus sequences of the active LINE-1 subfamilies in mice (Tf, A, and Gf), which were characterized in a previous study^4^, from Repbase^30,31^. Because they contained a variable number of monomer units, we tried to design primer pairs within the nonmonomer or truncated monomer region to avoid amplifying nonspecific sequences (Fig. 1 and Table 1). Because the consensus sequences were very similar among the A subfamilies (AI, AII, and AIII), the primers were designed to amplify all of these subfamilies.

### Sample preparation

Adult wild-type mice (C57BL/6) were used for collection of tissues, including frontal cortex, hippocampus, cerebellum, basal ganglia, heart, tail, testis, and ovary. All tissues were collected from female mice, except for the testis, which were collected from males (*N* = 3 for each tissue, unless otherwise described). The tissues were stored at −80 °C until use. The tissues were treated with 0.1 mg/ml of proteinase K (Roche) dissolved in DNA extraction buffer, and incubated at 55 °C overnight. Genomic DNA was extracted using the standard phenol–chloroform methods. All animal experiments were approved by the local animal experiment committees of RIKEN (Wako, Saitama, Japan). Animal experiments were carried out in accordance with the National Institutes of Health Guide for the Care and Use of Laboratory Animals. All efforts were made to minimize the number of animals used and their suffering.

Synthetic DNA samples were prepared to test the linearity and sensitivity of the assay. Sequences of fully-methylated (i.e. -CG-), and unmethylated (i.e. -TG-) were purchased from a manufacturer (FASMAC) (see Supplementary Table S1). These samples were mixed together to prepare different DNA methylation levels (0~100%, 10% intervals), and subjected to the assays as described below. Between the methylation range where we detected in the actual mouse samples (i.e. 50~70% for A and 50~80% for TfI and GfII), we prepared samples with 5% interval levels. Ninety-picograms, or two-hundred and ninety picograms of the mixed DNA samples were used in PCR for A, TfI and GfII subfamilies, respectively.

### Oxidative treatment and sodium bisulfite modification

Oxidative and sodium bisulfite treatments were performed using TrueMethyl Kit (Cambridge Epigenetix), according to the manufacturer’s instructions. In brief, 1.5-μg DNA samples were fragmented to approximately 5 kb by sonication (Covaris). Half the volume of the sample (equivalent to 750 ng of DNA) was subjected to oxidation followed by bisulfite modification (oxidative bisulfite sequencing, oxBS), and the other half was used for bisulfite modification only (bisulfite sequencing, BS). After purification using bead columns, both oxBS and BS samples were denatured with 50 mM NaOH at 37 °C for 30 min. One microliter of oxidation solution included in the kit was added to the oxBS sample, while 1 μl of water was added to BS sample. Samples were incubated at 40 °C for 30 min in a thermal cycler and then centrifuged for 10 min at 14,000 G. The supernatants were removed and directly underwent sodium bisulfite treatment following the manufacturer’s protocol. The samples were then desulfonated and washed with the provided buffers. All reagents and columns were included in the kit (Cambridge Epigenetix).

### Bisulfite PCR for pyrosequencing assay

One microliter of oxBS or BS DNA sample was used for PCR amplification. The reaction mixture contained the following reagents; 1× PCR amplification buffer (Invitrogen), 1 M betaine, 0.2 mM dNTP (TakaraBio), 3.0 mM MgCl_2_, 0.4 mM primers including biotin-labeled primer, 2.0 ng single-stranded DNA binding protein (Promega), and 5 U of Platinum *Taq* DNA polymerase (Invitrogen). PCR conditions were as follows: 3 min at 95 °C followed by 40 cycles of 10 s at 98 °C, 30 s at 58 °C, and 30 s at 72 °C for TfI and GfII; 3 min at 95 °C followed by 40 cycles of 10 s at 98 °C, 30 s at 55 °C, and 30 s at 72 °C for A. Primer sequences are listed in Table 1.

### Pyrosequencing

Pyrosequencing was performed as described previously^34^. Briefly, the PCR mixture was treated with 4 μl of streptavidin-sepharose beads (Amersham Biosciences) and 54 μl of 2× binding buffer (Qiagen). The samples were denatured with 0.2 N NaOH and washed thoroughly with washing buffer (Qiagen). The beads were then suspended in 50 μl of annealing buffer (Qiagen) containing 0.2 μM of the sequencing primers (Table 1), and the suspension was heated to 96 °C for 2 min. Pyrosequencing was performed using a Pyromark Gold Q96 Reagents kit (Qiagen) with a PSQ 96MA instrument (Qiagen) following the manufacturer’s protocols.

### TA cloning and Sanger sequencing

PCR products prepared as described above were subjected to TA-cloning using TOPO TA Cloning kit (Thermo Fisher Scientific) and One Shot TOP10 Chemically Competent *E. coli* (Thermo Fisher Scientific), according to the manufacturer’s instructions. After transformation, the single colonies were checked by colony PCR using M13 Forward (5′-GTAAAACGACGGCCAG-3′) and M13 Reverse primer (5′-CAGGAAACAGCTATGAC-3′). Sanger sequencing was then performed using T3 primer (5′-ATTAACCCTCACTAAAGGGA-3′) (Eurofins Genomics K.K.).

### Data analysis

The mC level was estimated from the oxBS sample by pyrosequencing. The hmC level was estimated by subtracting the oxBS level from the BS level. Statistical analysis was performed using Student’s t-test for the comparison of the two groups. For other tests, analysis of variance (ANOVA) followed by Tukey’s test was conducted. For correlation analysis, Pearson’s correlation test was used. For the purpose of comparison across different tissues, data from the four brain regions were averaged and treated as a single tissue. *P* < 0.05 was considered significant.

Sequence data of bisulfite PCR amplicon was processed using Sequencher (ver. 4.10.1., Gene Code Corporation) and aligned with the consensus sequences of the target subfamily using Genetyx (ver. 13, Genetyx Corporation). Phylogenetic trees were drawn using UPGMA method, implemented in Genetyx.

## Electronic supplementary material

Supplementary Information
