# Supplementary material for: DNA methylation and hydroxymethylation analyses of the active LINE-1 subfamilies in mice
Source: Sci Rep. 2017 Oct 19;7:13624. doi: 10.1038/s41598-017-14165-7 (PMC5648895; doi:10.1038/s41598-017-14165-7)
Supplement: Supplementary file 1 — Supplementary Information [file 41598_2017_14165_MOESM1_ESM.pdf]

DNA methylation and hydroxymethylation analyses of the active LINE-1 subfamilies in mice

Yui Murata[1], Miki Bundo[1,2], Junko Ueda[3], Mie Kubota-Sakashita[3], Kiyoto Kasai[4], Tadafumi Kato[3] and Kazuya Iwamoto[1*]

[1] Department of Molecular Brain Science, Graduate School of Medical Sciences, Kumamoto University, 1-1-1 Honjo, Chuo-ku, Kumamoto City, Kumamoto 860-8556, Japan.

[2] PRESTO, Japan Science and Technology Agency, Japan

[3] Laboratory for Molecular Dynamics of Mental Disorders, RIKEN Brain Science Institute, 2-1 Hirosawa, Wako-city, Saitama 351-0198, Japan.

[4] Department of Neuropsychiatry, Graduate School of Medicine, The University of Tokyo, 7-3-1 Hongo, Bunkyo-ku, Tokyo 113-8655, Japan.

[*] Corresponding author: Kazuya Iwamoto, Department of Molecular Brain Science, Kumamoto University, 1-1-1 Honjo, Kumamoto City, Kumamoto 860-8556, Japan. *Email address: iwamotok@kumamoto-u.ac.jp* (K. Iwamoto).

Supplementary Information

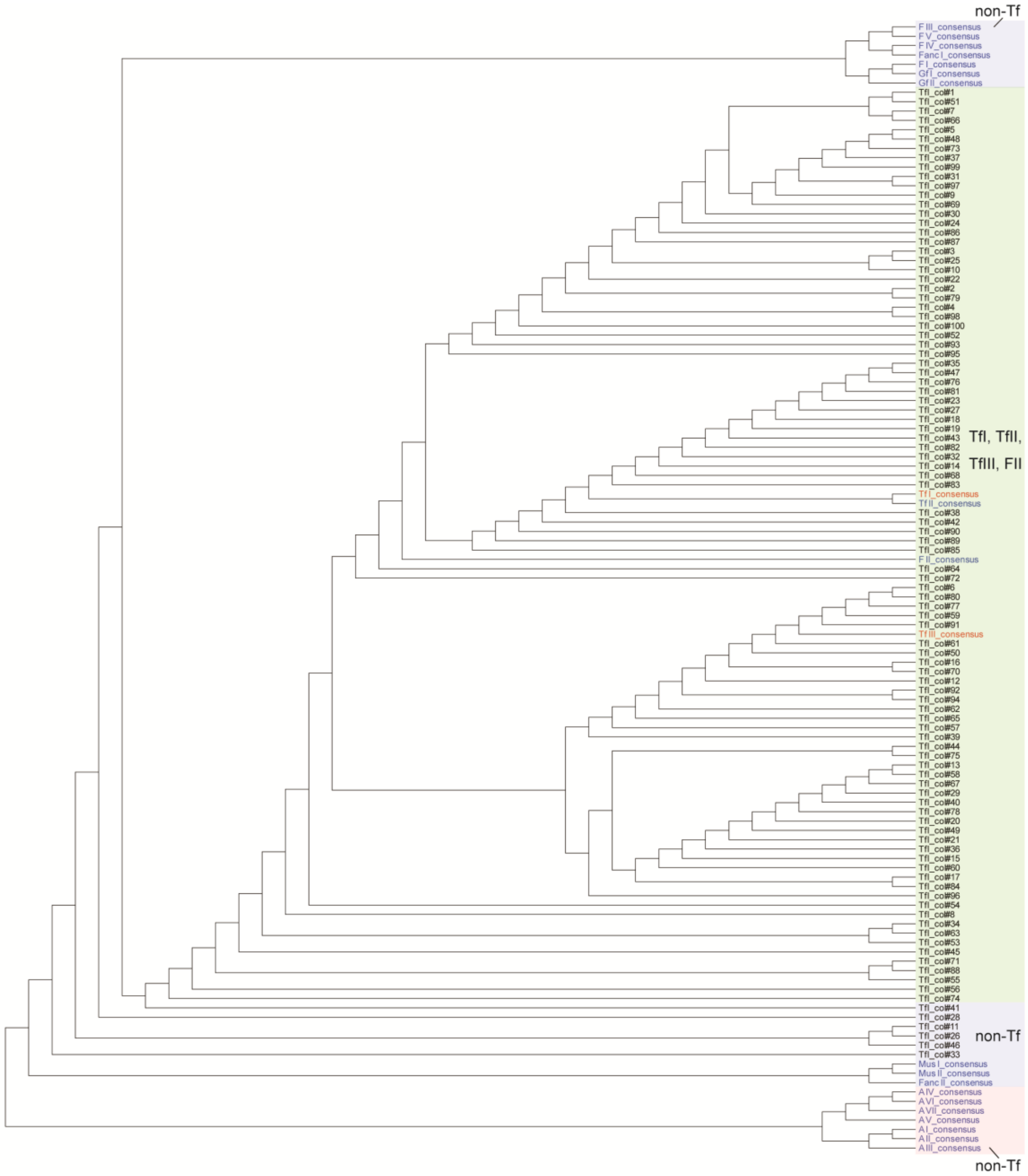

Supplementary Figure S1. Phylogenetic analysis for sequences derived from colonies with bisulfite-PCR product amplified for TfI. Bisulfite converted 5'-UTR regions of

LINE-1 consensus sequences were analyzed together. Out of 100 colonies, 95 were clustered with Tf or FII subfamilies. Close inspection of the sequences derived from colonies showed that all had TfI and TfIII-type variations. Sequencing primer for TfI further discriminates TfI from TfIII in the subsequent pyrosequecing reaction. Note that older subfamilies Lx, V, and nonhomologous subfamily, type N were not included. Samples in red are the target subfamilies, and those in blue are subfamilies not targeted by the assay.

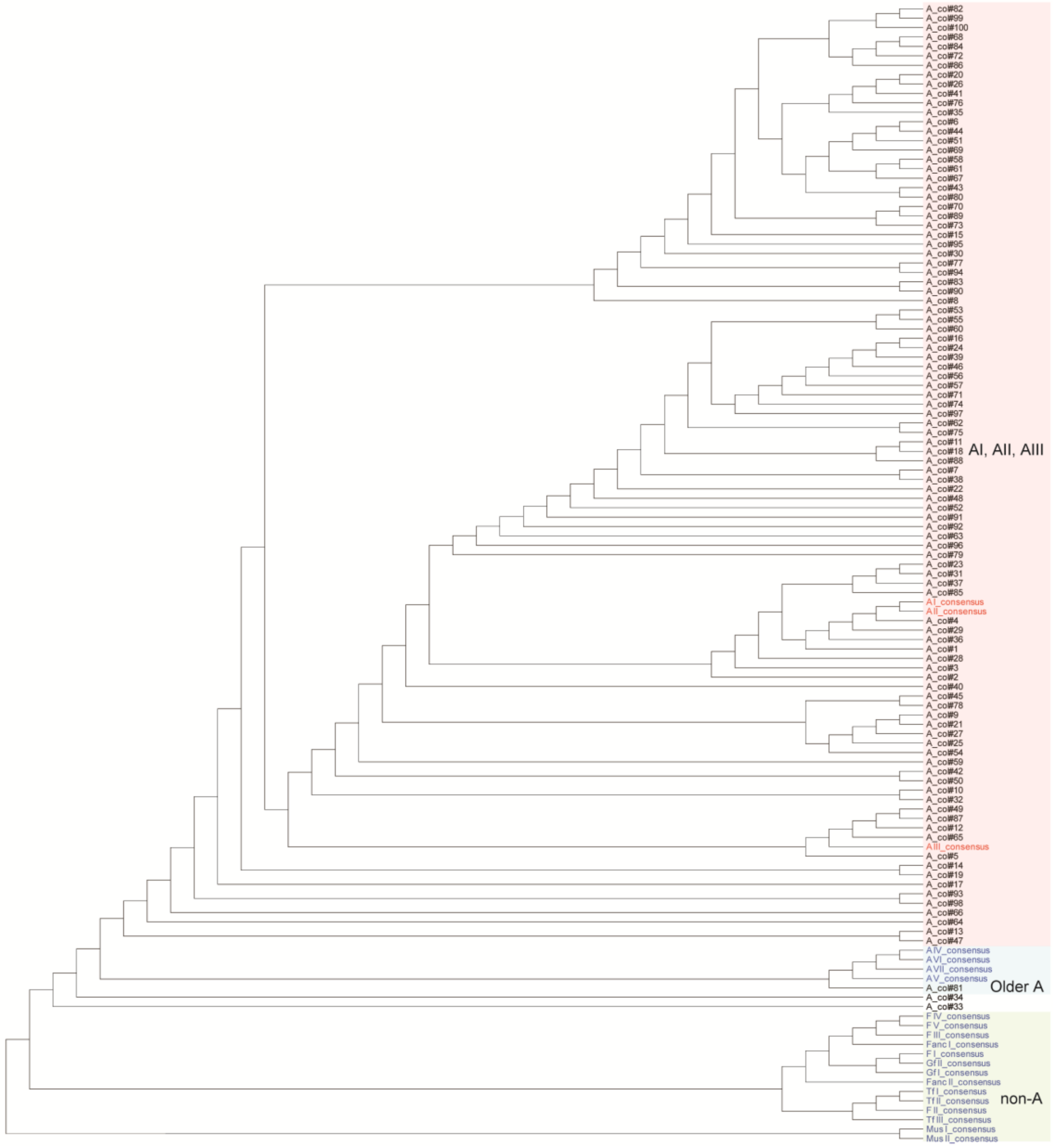

Supplementary Figure S2. Phylogenetic analysis for sequences derived from colonies with bisulfite-PCR product amplified for A (AI, AII and AIII). Bisulfite converted 5’-UTR regions of LINE-1 consensus sequences were analyzed together. Out of 100 colonies, 97 were clustered with types AI, AII or AIII. Note that older subfamilies Lx, V, and nonhomologous subfamily, type N were not included. Samples in red are the target

subfamilies, and those in blue are subfamilies not targeted by the assay.

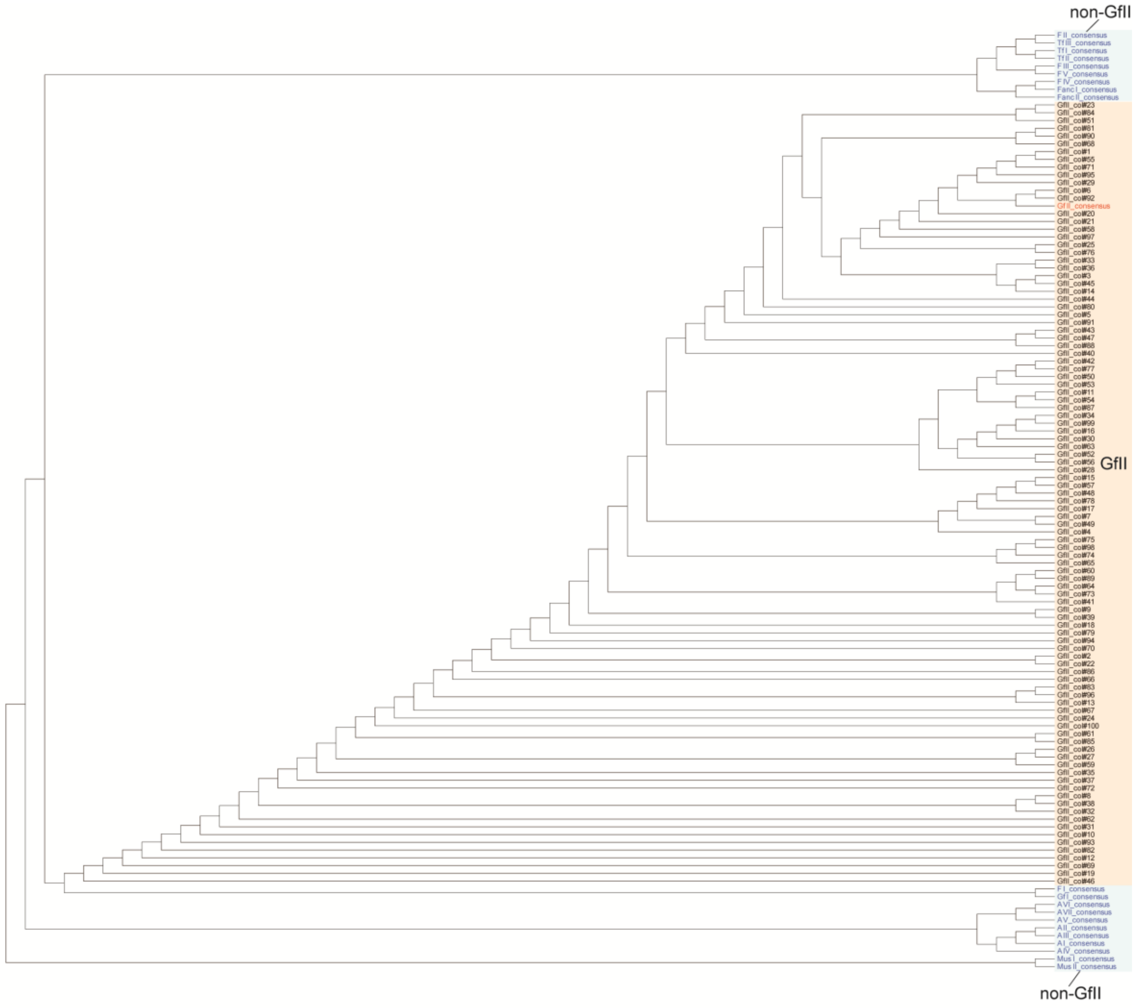

Supplementary Figure S3. Phylogenetic analysis for sequences derived from colonies with bisulfite-PCR product amplified for GfII. Bisulfite converted 5’-UTR regions of LINE-1 consensus sequences were analyzed together. All of the 100 colonies were clustered with GfII subfamilies. Note that older subfamilies Lx, V, and nonhomologous subfamily, type N were not included. Samples in red are the target subfamilies, and those in blue are subfamilies not targeted by the assay.

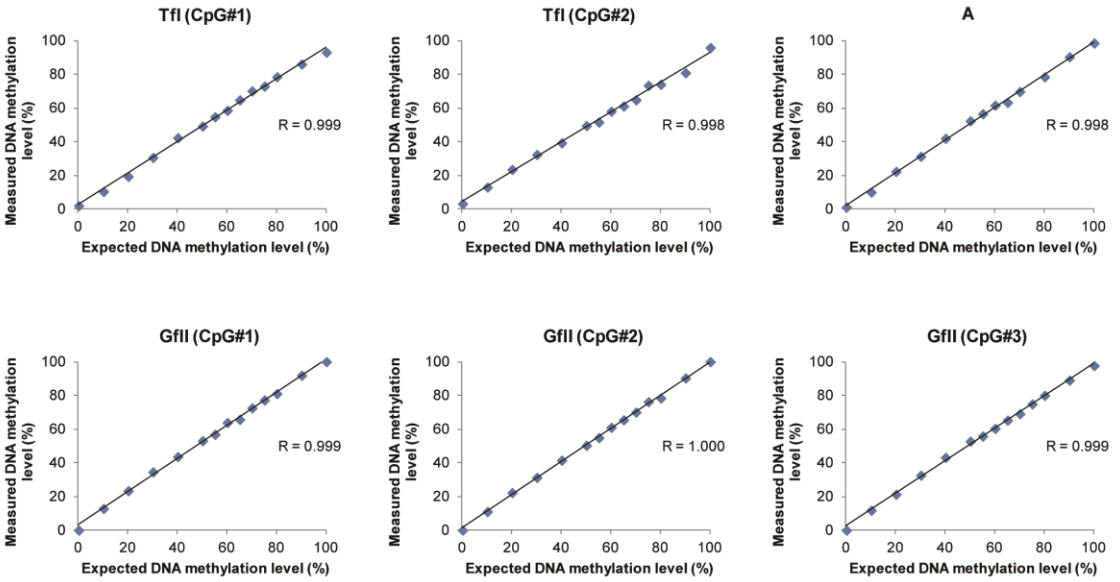

Supplementary Figure S4. Correlation between DNA methylation levels of synthetic DNA sequences. All of the CpG sites measured in the assays showed high correlations (R > 0.998).

Supplementary Table S1. Sequences of synthetic DNA.

Subfamily	Sample name	Sequence
A	AI_methylated	5’-gtgagtggaatataatttttgttaggagtttggtt**cg**aatattagatatttgggtatttgttttgtaagaagagagtttgtttgtagagaatattttgtttattgaaattaaggagagtgttatttttt-3’
	AI_unmethylated	5’-gtgagtggaatataatttttgttaggagtttggtt**tg**aatattagatatttgggtatttgttttgtaagaagagagtttgtttgtagagaatattttgtttattgaaattaaggagagtgttatttttt-3’
GfII	GfII_methylated	5’-gggggttattttgattttgggattt**cg**tag**cg**ggtagtttgtaggttaaagtaatatagtttttgggaaagattttgttttgggtttttatttt**cg**gttaggaggaggtttaaatattagataattgtgtatttttttgaaagaggagagtttgtttgtagagattgttttgattattgaaatttagggaagagagttagttttttggtttgttgatatagtgtaataaaattattagaggaa-3’
	GfII_unmethylated	5’-gggggttattttgattttgggattt**tg**tag**tg**ggtagtttgtaggttaaagtaatatagtttttgggaaagattttgttttgggtttttatttt**tg**gttaggaggaggtttaaatattagataattgtgtatttttttgaaagaggagagtttgtttgtagagattgttttgattattgaaatttagggaagagagttagttttttggtttgttgatatagtgtaataaaattattagaggaa-3’
TfI	TfI_methylated	5’-tttgggaattgttaaagtaatatagtgtttgagaaaggttttgttttgggtttttttttt**cg**gttaggaggaggtttaaatataagatatttg**cg**tattttttttgtaagagagtttgttagtagagagtgttttgagtattgaaatttagaggagagaatttgtttttttaggtttgttgataga**cg**gtaatagaattattagaagaataatttttaaatagagttaattataattattaattttagagattattagatgg-3’
	TfI_unmethylated	5’-tttgggaattgttaaagtaatatagtgtttgagaaaggttttgttttgggtttttttttt**tg**gttaggaggaggtttaaatataagatatttg**tg**tattttttttgtaagagagtttgttagtagagagtgttttgagtattgaaatttagaggagagaatttgtttttttaggtttgttgataga**tg**gtaatagaattattagaagaataatttttaaatagagttaattataattattaattttagagattattagatgg-3’
